# Supplementary material for: Modeling glioblastoma heterogeneity as a dynamic network of cell states
Source: Mol Syst Biol. 2021 Sep 16;17(9):e10105. doi: 10.15252/msb.202010105 (PMC8444284; doi:10.15252/msb.202010105)
Supplement: Supplementary file 5 — Source Data for Figure 3 [file MSB-17-e10105-s001.zip › Figure3A_sourcedata/GSEA_3065/nosigdb_state5.GseaPreranked.1622718435914/gsea_report_for_na_pos_1622718435914.html]

Report for na\_pos 1622718435914 [GSEA]

| GS  follow link to MSigDB | GS DETAILS | SIZE | ES | NES | NOM p-val | FDR q-val | FWER p-val | RANK AT MAX | LEADING EDGE || 1 | LARSSON\_STATE\_2 | Details ... | 85 | 1.00 | 3.93 | 0.000 | 0.000 | 0.000 | 100 | tags=100%, list=1%, signal=100% |
| 2 | LARSSON\_STATE\_3 | Details ... | 86 | 0.90 | 3.63 | 0.000 | 0.000 | 0.000 | 571 | tags=84%, list=6%, signal=89% |
| 3 | DOROSZKO\_U3180MG\_CLUSTER\_2 | Details ... | 463 | 0.70 | 3.33 | 0.000 | 0.000 | 0.000 | 1694 | tags=60%, list=19%, signal=70% |
| 4 | RICHARDS\_NOWAKOWSKI\_SCIENCE\_2017\_OPC\_UPREG | Details ... | 314 | 0.52 | 2.41 | 0.000 | 0.000 | 0.000 | 1174 | tags=38%, list=13%, signal=42% |
| 5 | ALLEN\_114\_OPCL1\_6MYT1 | Details ... | 16 | 0.84 | 2.24 | 0.000 | 0.000 | 0.000 | 990 | tags=75%, list=11%, signal=84% |
| 6 | RICHARDS\_ZHONG\_NATURE\_2018\_OPC\_UPREG | Details ... | 72 | 0.58 | 2.20 | 0.000 | 0.000 | 0.000 | 990 | tags=44%, list=11%, signal=50% |
| 7 | VANGRONINGEN\_MES | Details ... | 373 | 0.45 | 2.15 | 0.000 | 0.000 | 0.001 | 1418 | tags=38%, list=16%, signal=43% |
| 8 | IVY\_CTMVP | Details ... | 45 | 0.60 | 2.12 | 0.000 | 0.000 | 0.001 | 1177 | tags=44%, list=13%, signal=51% |
| 9 | NB\_KI\_CLUSTER8 | Details ... | 304 | 0.45 | 2.08 | 0.000 | 0.001 | 0.003 | 1899 | tags=42%, list=21%, signal=52% |
| 10 | RICHARDS\_NEFTEL\_CELL\_2019\_OPC | Details ... | 37 | 0.58 | 1.99 | 0.000 | 0.002 | 0.011 | 1022 | tags=51%, list=11%, signal=58% |
| 11 | RICHARDS\_NOWAKOWSKI\_SCIENCE\_2017\_ASTROCYTE\_UPREG | Details ... | 474 | 0.42 | 1.98 | 0.000 | 0.002 | 0.011 | 1305 | tags=34%, list=15%, signal=38% |
| 12 | GAROFANO\_IAVARONE\_NEU\_SUBTYPE | Details ... | 156 | 0.46 | 1.98 | 0.000 | 0.002 | 0.011 | 1714 | tags=39%, list=19%, signal=47% |
| 13 | SUVA\_OPC\_PMID\_31327527 | Details ... | 37 | 0.58 | 1.97 | 0.000 | 0.002 | 0.011 | 1022 | tags=51%, list=11%, signal=58% |
| 14 | DOROSZKO\_U3054MG\_CLUSTER\_5 | Details ... | 95 | 0.49 | 1.95 | 0.000 | 0.002 | 0.013 | 1999 | tags=43%, list=22%, signal=55% |
| 15 | ALLEN\_111\_ASTROL1\_6FGFR3ETNPPL | Details ... | 23 | 0.65 | 1.92 | 0.000 | 0.003 | 0.019 | 598 | tags=35%, list=7%, signal=37% |
| 16 | DOROSZKO\_U3054MG\_CLUSTER\_0 | Details ... | 56 | 0.53 | 1.90 | 0.000 | 0.004 | 0.030 | 473 | tags=34%, list=5%, signal=36% |
| 17 | NB\_KI\_CLUSTER2 | Details ... | 251 | 0.41 | 1.84 | 0.000 | 0.007 | 0.054 | 1258 | tags=28%, list=14%, signal=32% |
| 18 | LARSSON\_STATE\_5 | Details ... | 99 | 0.44 | 1.77 | 0.000 | 0.012 | 0.100 | 506 | tags=47%, list=6%, signal=50% |
| 19 | RICHARDS\_ZHONG\_NATURE\_2018\_ASTROCYTES\_UPREG | Details ... | 226 | 0.39 | 1.77 | 0.000 | 0.012 | 0.105 | 1075 | tags=32%, list=12%, signal=36% |
| 20 | ALLEN\_116\_OLIGOL4\_6OPALIN | Details ... | 20 | 0.60 | 1.75 | 0.011 | 0.013 | 0.113 | 1837 | tags=60%, list=20%, signal=75% |
| 21 | NB\_KI\_CLUSTER14 |  | 211 | 0.39 | 1.73 | 0.000 | 0.014 | 0.135 | 1510 | tags=30%, list=17%, signal=36% |
| 22 | IVY\_CT |  | 144 | 0.41 | 1.73 | 0.000 | 0.014 | 0.136 | 1103 | tags=24%, list=12%, signal=27% |
| 23 | NB\_KI\_CLUSTER17 |  | 126 | 0.41 | 1.73 | 0.000 | 0.013 | 0.137 | 961 | tags=36%, list=11%, signal=39% |
| 24 | TIISIG\_PMID\_32649888 |  | 23 | 0.58 | 1.73 | 0.013 | 0.013 | 0.141 | 1294 | tags=39%, list=14%, signal=46% |
| 25 | NB\_KI\_CLUSTER6 |  | 468 | 0.35 | 1.69 | 0.000 | 0.016 | 0.182 | 2354 | tags=41%, list=26%, signal=52% |
| 26 | NB\_KI\_CLUSTER7 |  | 262 | 0.37 | 1.69 | 0.000 | 0.017 | 0.189 | 2607 | tags=36%, list=29%, signal=50% |
| 27 | NB\_KI\_CLUSTER23 |  | 246 | 0.38 | 1.68 | 0.000 | 0.017 | 0.197 | 1125 | tags=31%, list=13%, signal=35% |
| 28 | DOROSZKO\_U3054MG\_CLUSTER\_2 |  | 233 | 0.36 | 1.65 | 0.000 | 0.019 | 0.230 | 2069 | tags=41%, list=23%, signal=52% |
| 29 | RICHARDS\_NOWAKOWSKI\_SCIENCE\_2017\_NEN\_EARLY2\_UPREG |  | 388 | 0.35 | 1.65 | 0.000 | 0.018 | 0.230 | 1729 | tags=31%, list=19%, signal=37% |
| 30 | OLSEN\_SCPLIKE |  | 78 | 0.42 | 1.61 | 0.000 | 0.025 | 0.301 | 670 | tags=36%, list=7%, signal=38% |
| 31 | DEPRETER\_LOW\_NB\_RISK |  | 18 | 0.58 | 1.61 | 0.023 | 0.025 | 0.315 | 748 | tags=39%, list=8%, signal=42% |
| 32 | DOROSZKO\_U3054MG\_CLUSTER\_7 |  | 27 | 0.51 | 1.59 | 0.017 | 0.029 | 0.360 | 1823 | tags=33%, list=20%, signal=42% |
| 33 | KRONA\_ROSEN\_WHITE\_MATTER\_INVASION |  | 180 | 0.36 | 1.57 | 0.007 | 0.031 | 0.398 | 1856 | tags=35%, list=21%, signal=43% |
| 34 | ALLEN\_112\_ASTROL1FGFR3MT1G |  | 25 | 0.51 | 1.57 | 0.032 | 0.031 | 0.406 | 754 | tags=40%, list=8%, signal=44% |
| 35 | DOROSZKO\_U3013MG\_CLUSTER\_0 |  | 27 | 0.50 | 1.56 | 0.020 | 0.032 | 0.421 | 684 | tags=41%, list=8%, signal=44% |
| 36 | GANDAL\_ALCOHOLADDICTION\_DN\_PMID29439242 |  | 57 | 0.42 | 1.55 | 0.011 | 0.032 | 0.426 | 1559 | tags=39%, list=17%, signal=46% |
| 37 | ALLEN\_113\_ASTROL1FGFR3FOS |  | 24 | 0.50 | 1.53 | 0.028 | 0.036 | 0.471 | 758 | tags=42%, list=8%, signal=45% |
| 38 | DOROSZKO\_U3054MG\_CLUSTER\_1 |  | 16 | 0.57 | 1.53 | 0.045 | 0.036 | 0.486 | 831 | tags=31%, list=9%, signal=34% |
| 39 | RICHARDS\_NEFTEL\_CELL\_2019\_AC |  | 32 | 0.47 | 1.52 | 0.029 | 0.037 | 0.502 | 663 | tags=44%, list=7%, signal=47% |
| 40 | RICHARDS\_NOWAKOWSKI\_SCIENCE\_2017\_VRG\_UPREG |  | 380 | 0.32 | 1.52 | 0.000 | 0.036 | 0.503 | 1618 | tags=34%, list=18%, signal=40% |
| 41 | SUVA\_AC\_PMID\_31327527 |  | 32 | 0.47 | 1.51 | 0.028 | 0.038 | 0.527 | 663 | tags=44%, list=7%, signal=47% |
| 42 | NB\_KI\_CLUSTER24 |  | 171 | 0.35 | 1.50 | 0.000 | 0.041 | 0.555 | 3097 | tags=44%, list=34%, signal=67% |
| 43 | NB\_KI\_CLUSTER20 |  | 16 | 0.55 | 1.50 | 0.055 | 0.042 | 0.569 | 1076 | tags=38%, list=12%, signal=43% |
| 44 | OUTERRADIALGLIA |  | 48 | 0.42 | 1.49 | 0.017 | 0.042 | 0.580 | 741 | tags=29%, list=8%, signal=32% |
| 45 | ALMSTEDT\_ALK |  | 48 | 0.42 | 1.49 | 0.023 | 0.041 | 0.582 | 1172 | tags=23%, list=13%, signal=26% |
| 46 | POLLEN\_OUTERRADIALGLIA\_PMID\_26406371 |  | 48 | 0.42 | 1.49 | 0.018 | 0.042 | 0.596 | 741 | tags=29%, list=8%, signal=32% |
| 47 | RICHARDS\_VERHAAK\_CANCERCELL\_2010\_CLASSICAL |  | 112 | 0.35 | 1.47 | 0.009 | 0.046 | 0.636 | 1822 | tags=39%, list=20%, signal=49% |
| 48 | SEGERMAN\_MULTITHERAPY\_RESISTANCE\_FIG5A\_PMID\_27974212 |  | 24 | 0.48 | 1.46 | 0.055 | 0.047 | 0.648 | 2768 | tags=67%, list=31%, signal=96% |
| 49 | GANDAL\_BIPOLAR\_UP\_PMID29439242 |  | 58 | 0.39 | 1.44 | 0.020 | 0.054 | 0.709 | 1520 | tags=33%, list=17%, signal=39% |
| 50 | DOROSZKO\_PERIVASCULAR\_INVASION |  | 34 | 0.43 | 1.40 | 0.056 | 0.065 | 0.778 | 549 | tags=29%, list=6%, signal=31% |
| 51 | RICHARDS\_NOWAKOWSKI\_SCIENCE\_2017\_MGE\_RG1\_UPREG |  | 310 | 0.29 | 1.38 | 0.000 | 0.074 | 0.821 | 1211 | tags=25%, list=13%, signal=28% |
| 52 | NB\_KI\_CLUSTER15 |  | 155 | 0.32 | 1.37 | 0.006 | 0.078 | 0.842 | 2628 | tags=34%, list=29%, signal=47% |
| 53 | RICHARDS\_NOWAKOWSKI\_SCIENCE\_2017\_IPC\_NEN1\_UPREG |  | 226 | 0.30 | 1.36 | 0.000 | 0.080 | 0.855 | 1766 | tags=36%, list=20%, signal=43% |
| 54 | RICHARDS\_NOWAKOWSKI\_SCIENCE\_2017\_IN\_CTX\_CGE2\_UPREG |  | 224 | 0.30 | 1.36 | 0.008 | 0.079 | 0.856 | 883 | tags=20%, list=10%, signal=22% |
| 55 | SINGH\_SOX2\_UPREGULATED\_TRANSCRIPTS\_PMID\_28122245 |  | 410 | 0.29 | 1.36 | 0.000 | 0.080 | 0.867 | 1921 | tags=29%, list=21%, signal=35% |
| 56 | WANG\_CLASSICAL\_PMID\_28697342 |  | 36 | 0.41 | 1.36 | 0.077 | 0.080 | 0.869 | 1769 | tags=36%, list=20%, signal=45% |
| 57 | GANDAL\_IBD\_UP\_PMID29439242 |  | 56 | 0.36 | 1.33 | 0.047 | 0.091 | 0.907 | 1625 | tags=38%, list=18%, signal=45% |
| 58 | RICHARDS\_VERHAAK\_CANCERCELL\_2010\_PRONEURAL |  | 110 | 0.32 | 1.32 | 0.026 | 0.095 | 0.920 | 1684 | tags=33%, list=19%, signal=40% |
| 59 | RICHARDS\_NOWAKOWSKI\_SCIENCE\_2017\_IN\_STR\_UPREG |  | 174 | 0.29 | 1.22 | 0.054 | 0.167 | 0.991 | 1241 | tags=25%, list=14%, signal=28% |
| 60 | ANASTASSIOU\_MULTICANCER\_INVASIVENESS\_PMID\_22208948 |  | 27 | 0.40 | 1.22 | 0.176 | 0.173 | 0.992 | 1824 | tags=63%, list=20%, signal=79% |
| 61 | GANDAL\_SCHIZOPHRENIA\_UP\_PMID29439242 |  | 74 | 0.31 | 1.20 | 0.135 | 0.186 | 0.995 | 756 | tags=23%, list=8%, signal=25% |
| 62 | ALLEN\_115\_OLIGOL4\_6MOBPCOL18A1 |  | 23 | 0.40 | 1.19 | 0.202 | 0.200 | 0.999 | 1837 | tags=52%, list=20%, signal=65% |
| 63 | PARADA\_T1\_DN\_26461091 |  | 39 | 0.34 | 1.17 | 0.174 | 0.209 | 0.999 | 2019 | tags=36%, list=22%, signal=46% |
| 64 | RICHARDS\_ZHONG\_NATURE\_2018\_MICROGLIA\_UPREG |  | 196 | 0.26 | 1.17 | 0.076 | 0.214 | 1.000 | 1047 | tags=21%, list=12%, signal=24% |
| 65 | ANGIOGENESIS\_GO\_0001525 |  | 20 | 0.39 | 1.15 | 0.242 | 0.236 | 1.000 | 546 | tags=20%, list=6%, signal=21% |
| 66 | KRONA\_ROSEN\_PERIVASCULAR\_INVASION |  | 43 | 0.32 | 1.10 | 0.278 | 0.313 | 1.000 | 2037 | tags=40%, list=23%, signal=51% |
| 67 | DOROSZKO\_U3180MG\_CLUSTER\_1 |  | 15 | 0.41 | 1.09 | 0.374 | 0.319 | 1.000 | 990 | tags=33%, list=11%, signal=37% |
| 68 | TISIG\_PMID\_32649888 |  | 34 | 0.32 | 1.07 | 0.331 | 0.343 | 1.000 | 1670 | tags=35%, list=19%, signal=43% |
| 69 | RICHARDS\_LIDDELOW\_NATURE\_2017\_A1\_REACTIVE\_ASTROCYTES |  | 20 | 0.37 | 1.06 | 0.371 | 0.372 | 1.000 | 1571 | tags=40%, list=17%, signal=48% |
| 70 | ALMSTEDT\_RISK |  | 30 | 0.32 | 1.03 | 0.402 | 0.434 | 1.000 | 748 | tags=23%, list=8%, signal=25% |
| 71 | DOROSZKO\_U3013MG\_CLUSTER\_1 |  | 36 | 0.30 | 1.00 | 0.444 | 0.502 | 1.000 | 825 | tags=19%, list=9%, signal=21% |
| 72 | DOROSZKO\_U3054MG\_CLUSTER\_3 |  | 195 | 0.22 | 0.99 | 0.464 | 0.514 | 1.000 | 1826 | tags=25%, list=20%, signal=30% |
| 73 | DOROSZKO\_U3054MG\_CLUSTER\_10 |  | 241 | 0.21 | 0.97 | 0.540 | 0.555 | 1.000 | 1263 | tags=19%, list=14%, signal=22% |
| 74 | NB\_KI\_CLUSTER9 |  | 21 | 0.30 | 0.89 | 0.621 | 0.737 | 1.000 | 2727 | tags=38%, list=30%, signal=55% |
| 75 | NB\_KI\_CLUSTER22 |  | 133 | 0.19 | 0.81 | 0.951 | 0.879 | 1.000 | 1736 | tags=23%, list=19%, signal=28% |
| 76 | PARADA\_T2\_DN\_26461091 |  | 39 | 0.24 | 0.79 | 0.837 | 0.894 | 1.000 | 1796 | tags=28%, list=20%, signal=35% |
| 77 | GANDAL\_BIPOLAR\_DN\_PMID29439242 |  | 17 | 0.28 | 0.78 | 0.806 | 0.897 | 1.000 | 571 | tags=18%, list=6%, signal=19% |
| 78 | GANDAL\_SCHIZOPHRENIA\_DN\_PMID29439242 |  | 16 | 0.19 | 0.53 | 0.981 | 0.996 | 1.000 | 2336 | tags=25%, list=26%, signal=34% |
Table: Gene sets enriched in phenotype **na **[plain text format]****
